# Prognostic Significance of EDN/RB, HJURP, p60/CAF-1 and PDLI4, Four New Markers in High-Grade Gliomas

**DOI:** 10.1371/journal.pone.0073332

**Published:** 2013-09-11

**Authors:** Marie de Tayrac, Stephan Saikali, Marc Aubry, Pascale Bellaud, Rachel Boniface, Véronique Quillien, Jean Mosser

**Affiliations:** 1 Centre National de la Recherche Scientifique (CNRS), Unité mixte de recherche – UMR 6290, Institut Génétique et Développement de Rennes, Rennes, France; 2 Université Rennes 1, UEB - Université européenne de Bretagne, Biosit, Faculté de Médecine, Rennes, France; 3 CHU - Centre Hospitalier universitaire de Rennes, Service de Génétique Moléculaire et Génomique, Rennes, France; 4 CHU - Centre Hospitalier universitaire de Rennes, Service d'anatomie et cytologie pathologiques, Rennes, France; 5 Plate-forme Génomique Santé Biogenouest, Biosit, Rennes, France; 6 Plate-forme Histo-Pathologie, Biosit, Rennes, France; 7 Département de Biologie Médicale, Centre Eugène Marquis, Rennes, France; Beijing Tiantan Hospital, Capital Medical University, China

## Abstract

**Background:**

Recent studies have highlighted the heterogeneity of gliomas and demonstrated that molecular and genetic analysis could help in their classification and in the design of treatment protocols. In a previous study we have identified a 4-gene signature highly correlated with survival of glioma patients. The aim of this study is to confirm and extend these findings by investigating the expression of these genes at the protein level and their association with outcome of patients with high grade gliomas.

**Methodology/Principal Findings:**

Immunohistochemical staining for EDN/RB, HJURP, p60/CAF-1 and PDLI4 was studied on archive materials from 96 patients (64 glioblastomas and 32 grade III gliomas). The levels of all four proteins differed significantly between grade **III** and grade IV tumours. The levels of the EDN/RB, HJURP and p60/CAF-1 proteins were strongly associated with overall survival (p<0.001, p<0.001 and p=0.002, respectively), whereas the one of PDLI4 was not (P=0.11). A risk criterion defined as high levels of at least two of the EDN/RB, HJURP and p60/CAF-1 proteins accurately predicted the prognosis of patients. Multivariate analysis confirmed that this criterion was an independent negative prognostic marker (hazard ratio = 2.225; 95% CI, 1.248 to 3.966, p=0.007).

**Conclusions:**

The expression of the EDN/RB, HJURP, p60/CAF-1 and PDLI4 proteins is disrupted in high grade gliomas and increases in the levels of these proteins are closely linked to tumour aggressiveness and poor outcome.

## Introduction

High-grade gliomas (HGGs) are the most frequent and aggressive primary tumours of the brain. They have a dense cellularity and a high proliferation index, and display microvascular proliferation and/or necrosis [1]. HGG diagnosis is based on biopsy or tumour resection, according to the revised World Health Organization (WHO) classification. HGGs comprise grade III gliomas of various histological profiles [anaplastic astrocytoma (AA), mixed anaplastic oligoastrocytoma (AOA) and anaplastic oligodendroglioma (AO)] and grade IV gliomas (glioblastoma). Tumour grade is the most informative factor for stratification into subgroups with different prognoses. Glioblastoma has the worst prognosis, with a median overall survival of only 15 months, whereas grade III gliomas have a median overall survival of four years.

One major limitation of the WHO classification for HGGs is that the diagnosis of these tumours is particularly challenging and misclassification is therefore highly likely. Indeed, HGGs often display intratumoral morphological heterogeneity, making diagnosis difficult and often leading to inter-observer variability. It was recently reported that the concordance between local diagnosis and central neuropathology review may be as low as 50% [2]. Thus, the identification of biomarkers predictive of the outcome of patients would be a key way to improve the diagnosis of HGGs.

Only a few molecular markers have proved reliable and useful in clinical practice to date. The most used widely used are GFAP for assessing glial differentiation and Ki67/MIB1 for assessing proliferation, both of which are detected with antibodies. However, these antibodies cannot resolve the problems relating to HGG diagnosis. *MGMT* promoter methylation and *IDH1* mutational status have more recently been proposed as molecular markers for HGGs and have been shown to be highly correlated with patient outcome. As paraffin-embedded tumour samples are generally available at the time of diagnosis, the determination of these biomarkers by immunohistochemistry has been suggested. However, such a detection method for MGMT is not standardised and lacks reproducibility and correlation with clinical outcome [3,4]. For IDH1, the development of a monoclonal antibody that specifically and sensitively recognises the IDH1 protein carrying the R132H mutation has recently been reported [5]. Further evaluation of the anti-R132H antibody is required, but it seems likely that the use of this antibody will improve the diagnosis of gliomas.

Many recent investigations have attempted to identify new biomarkers for gliomas classification and prognostication through microarray analyses of gene expression. We recently described a prognostic classification for HGGs based on the levels of mRNA for four genes: *EDNRB*, *HJURP*, *CHAF1B* and *PDLIM4* [6]. These genes were identified in a meta-analysis of gene expression as being highly correlated to both HGG grade and survival. The prognostic value of this genetic classifier compared favourably with those of the mutational status of the *IDH1* gene and the methylation status of the *MGMT* promoter.

In this study, we investigated the levels of the EDN/RB, HJURP, p60/CAF-1 and PDLI4 proteins in HGGs. We provide evidence that the levels of these proteins are significantly correlated with histological grade and survival in glioma patients. We also highlight the prognostic value of integrating immunohistological data for three of these proteins (EDN/RB, HJURP and p60/CAF-1) into the diagnosis of HGGs.

## Materials and Methods

### Ethics Statement

All patients involved in this study provided written consent for the use of tissue samples for research proposals. Sample collection (n° AC-2008-77) was stored by the CRB tissue bank of Rennes (http://www.crbsante-rennes.com/index-1200.html). The consent procedure and research investigation performed with the human samples were approved by the human Ethics Committee of Rennes University Medical School and Hospital and were conducted according to the principals of the Declaration of Helsinki.

### Patients and Tissue Specimens

This study was conducted on 96 consecutive patients admitted to the Neurosurgical Department of Rennes University Hospital for surgical procedures for histologically diagnosed HGG, from 1999 to 2006. This cohort of patients included 64 cases of glioblastoma (grade IV) and 32 cases of grade III glioma (24 AA and 8 AO). All initial histological specimens were reviewed by a single neuropathologist (blind to the data for the patient) for confirmation of the original diagnosis in accordance with the WHO classification of central nervous system tumours. The clinical data recorded included age at diagnosis, sex and preoperative performance status. The MRI postoperative exam was done within 72 hours after surgery in order to avoid edema and neovessel formation which might cause enhancement that could be misdiagnosed as residual tumor. Patients underwent subtotal or gross total resection. Total excision was considered to have been achieved when no residual enhancement was observed on the postoperative control MRI. Overall survival was measured from the date of surgery until death or last clinical examination, up to July 1^st^ 2009. None of the patients developed leptomeningeal dissemination or distant metastasis. The clinical information is detailed in Table 1. Six normal brain tissue samples were collected during post mortem from donors who died of non-neurological diseases.

**Table 1:** Clinical characteristics of the patients and univariate survival analysis.

			All patients	Survival
Characteristic			(N=96)	Univariate analysis
Age--no.				p=0.03
	≤50 years		28	
	>50 years		68	
Sex--no.				NS
	Male		51	
	Female		45	
Preoperative KPS performance status –no.				NS
	≤ 70		44	
	> 70		46	
	ND		6	
Extent of surgery — no.				NS
	Debulking			
		Partial resection	25	
		Complete resection	67	
	ND		4	
Treatment(*)—no.				p=0.01
	None		2	
	Radiotherapy alone		16	
	Chemotherapy alone		3	
	Radiotherapy plus chemotherapy			
		Temozolomide	35	
		PCV	16	
		Other	22	
	ND		2	
Findings on pathological review — no.				p = 6 x 10^-5^
	WHO grade IV (GBM)		64	
	WHO grade III (24 AA and 8 AO)		32	
Cytoplasmic EDNRB - (%)				p < 0.001
	Median		81	
	Range		12--100	
Nuclear p60/CAF-1 – (%)				p < 0.001
	Median		25	
	Range		4--60	
Nuclear HJURP - (%)				p=0.002
	Median		10	
	Range		0--34	
Cytoplasmic PDLI4 - (%)				p=0.11
	Median		50	
	Range		4--91	
Survival -- months				
	Median		16	
	95CI		14-19.1	

(*) PCV consists of three chemotherapy drugs: procarbazine, CCNU and vincristine. Other: includes topotecan, BCNU, Gemini and 8-drug chemotherapy

### Immunohistochemical Procedure

Immunohistochemistry was performed on 4 µm sections cut from formalin-fixed, paraffin-embedded gliomas. Sections were subjected to routine deparaffinisation, rehydration and the blocking of endogenous peroxidase activity, and antigen retrieval was carried out by immersion in 0.01 M sodium citrate buffer (pH 6.0) for 40 min in an 80°C water bath. Endogenous peroxidase activity was quenched by incubation with 10% H_2_O_2_ in PBS for 20 minutes. The monoclonal mouse anti-human clone SS 53 (Abcam), and clone 8Z11 (IBL) antibodies were used to study p60/CAF-1 and EDN/RB, respectively. The monoclonal rabbit anti-human antibodies HPA011912 (Sigma), and product number HPA008436 (SIGMA) were used to study PDLI4 and HJURP, respectively. Primary antibodies were diluted in 10% serum in PBS and incubated with the sections overnight, at 4°C, in a humid chamber (dilutions of 1:500, 1:50, 1:500 and 1:100 for p60/CAF-1, PDLI4, HJURP and EDN/RB, respectively, in the antibody diluent of the Dako Cytomation kit (Trappes, France)). Tumour sections were stained with the Vectastain kit (Vector, Burlingame, USA) and biotinylated with the RTU Vectastain Elite ABC kit (Vector), according to the manufacturer’s instructions. Antibody binding was detected with the peroxidase substrate kit (Vector), with haematoxylin counterstaining.

### Control Materials

External positive controls were used for each type of staining: breast adenocarcinoma for p60/CAF-1, normal striated muscle for PDLI4, normal liver for HJURP and lung adenocarcinoma for EDN/RB. Negative controls were obtained by omitting the primary antibody.

### Immunohistochemical quantification

Sections were examined under a Leitz-Diaplan microscope (Nuremburg, Germany). The percentage of cells displaying immunoreactivity (nuclear staining for p60/CAF-1 and HJURP and cytoplasmic staining for PDLI4 and EDN/RB) was recorded for each staining procedure, based on counts obtained at high magnification (x1000), for 500 tumour cells in two different, highly immunoreactive areas. Positive and negative controls were used to confirm the adequacy of staining for each protein. All tissue specimens were evaluated by a neuropathologist and a biologist blind to the clinical data for the patient.

### IDH1 Mutations

Exon 4 of the *IDH1* gene was amplified by PCR assay from tumour sample DNA and sequenced, as previously described [7]. Patients for whom DNA samples were available (n=75) were screened for somatic mutations affecting the R132 residue of IDH1.

### MGMT Promoter Methylation

The pyrosequencing methylation assay was performed with the PyroMark Q96 CpG MGMT kit (Qiagen), according to the manufacturer’s protocol. Samples were considered methylated if they had average CpG methylation ≥ 9% and unmethylated if they had average methylation <9%, in duplicate reactions.

### Statistical methods

All statistical analyses were carried out with the R statistical environment and packages (http://www.R-project.org/). The Wilcoxon rank sum test was used to analyse the relationship between protein levels (percentage of cells stained) and mRNA levels, and clinical and pathological characteristics.

#### Selection of Cut-off Scores

Clinically important cut-off scores for each protein were selected on the basis of time-dependent ROC curve analysis. This analysis was performed with R software and with the survival ROC package. The prognostic value of each of the markers was assessed by plotting the cumulative AUC over time curve (Table 2). The time point for which survival prediction was most accurate was then identified and the 95% confidence interval (CI) for the AUC at that time point was obtained by 500 bootstrap replications of the data. The ROC curve for the marker at the time of greatest predictive accuracy was plotted and used to identify the optimal immunohistochemical cut-off score. The optimal cut-off score was selected by identifying the point on the curve closest to the point (0,1) or the upper-left hand corner of the ROC curve plot (Figure S1).

**Table 2:** Prognostic accuracy of the four markers by time-dependent ROC curve analyses.

Marker	Peak accuracy (months)	AUC (95%)	Cut-off (%)	Sensitivity	Specificity	PPV(*)
EDN/RB	39 to 55	0.68 (0.57-0.78)	80	0.59	0.77	0.72
p60/CAF-1	21 to 27	0.69 (0.58-0.79)	24	0.69	0.69	0.69
HJURP	28 to 29	0.69 (0.59-0.79)	6	0.92	0.46	0.63
PDLI4	39 to 55	0.65 (0.53-0.78)	20	0.86	0.44	0.61

(*) PPV : positive predictive value

#### Survival Analysis

Univariate survival analyses were performed to estimate the effect of the clinical parameters and EDN/RB, HJURP, p60/CAF-1 and PDLI4 levels. Kaplan-Meier survival curves for both low and high levels of protein were analysed by log-rank tests with the selected cut-off point. Cox analysis was used to determine significance levels for each protein in a multivariate model including patient age and treatment, with the aim of identifying a set of independent prognostic factors. For the combined study of EDN/RB, HJURP and p60/CAF-1, risk evaluation was performed separately for each protein to provide risk categories weighted as 0 for low-risk and 1 for high-risk. These weights were summed over the three proteins to obtain a risk factor with four ordered risk levels (0 to 3). Patients were grouped into two categories according to this risk factor: patients with a risk factor ≥ 2 were grouped into a high-risk category and patients with a risk factor < 2 were grouped into low-risk category. Survival analyses were carried out with the R package Survival.

## Results

### Levels of EDN/RB, HJURP, p60/CAF-1 and PDLI4 in non-tumoral brain

**Figure 1:**
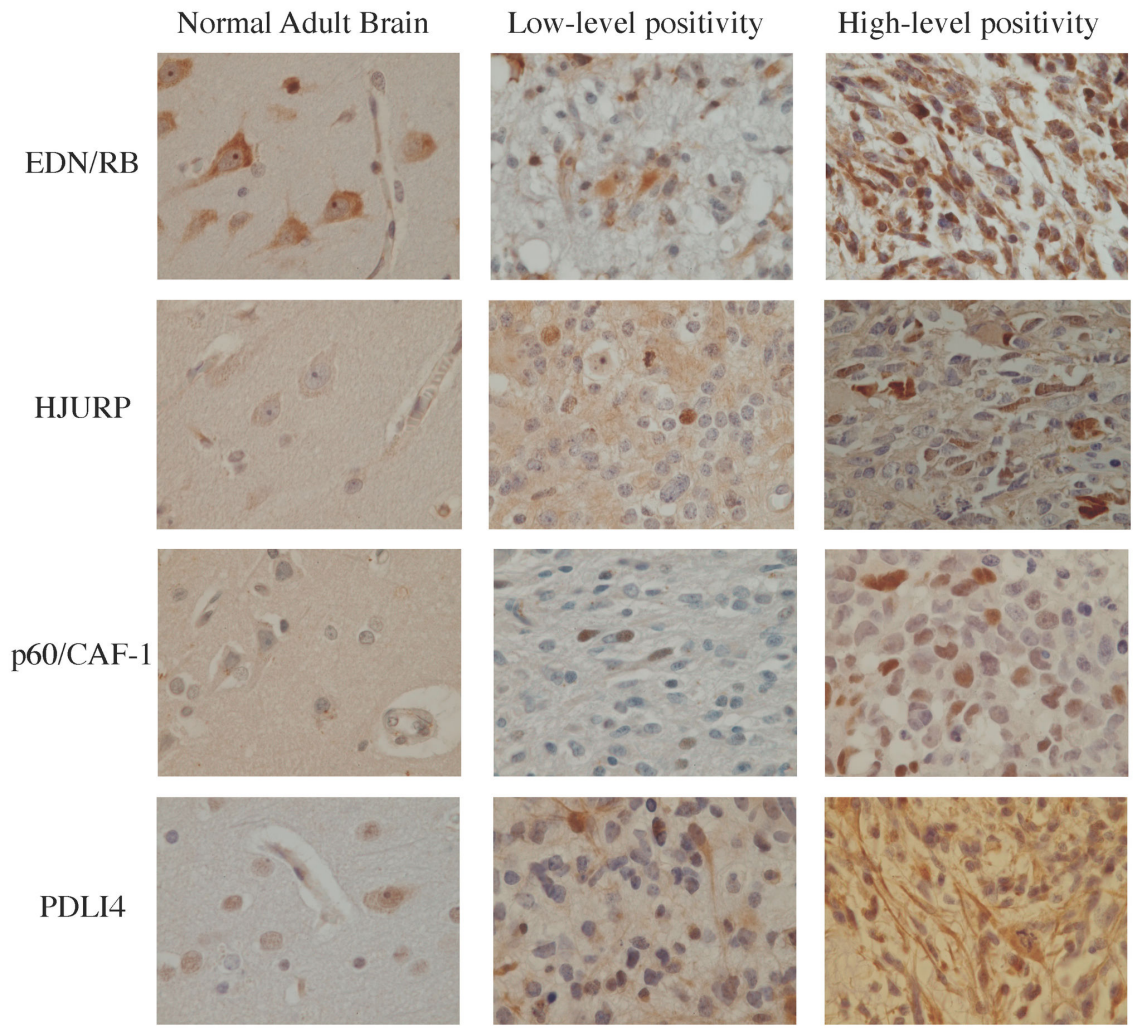
Examples of the range of marker immunopositivity within normal adult brain and high-grade gliomas. Sections of paraffin-embedded specimens from six normal brain tissues and 96 HGG specimens, consisting of WHO grade III and IV glioma samples, were stained by immunohistochemistry with antibodies against EDN/RB, HJURP, p60/CAF-1 and PDLI4. Representative data are reported for each protein: a section of normal adult brain tissue, a section from a weakly positive tumour and a section from a strongly positive tumour.

We evaluated the levels of the EDN/RB, HJURP, p60/CAF-1 and PDLI4 proteins by immunohistochemistry in six adult brain samples, four foetal brain samples and 96 high-grade glioma samples, including 64 glioblastomas (grade IV) and 32 grade III gliomas (24 AA and 8 AO). No immunoreactivity for the p60/CAF-1 protein was observed in adult brain, whereas nuclear immunoreactivity for this protein was observed in foetal oligodendrocytes. EDN/RB immunoreactivity was detected in the cytoplasm of neurons and endothelial cells in both adult and foetal brains and nuclear immunoreactivity for this protein was observed in foetal oligodendrocytes. HJURP immunoreactivity was observed in the nucleus of oligodendrocytes and in the cytoplasm of both neurons (adult brain) and endothelial cells (adult and foetal brain). We detected PDLI4 immunoreactivity in the nucleus of astrocytes and the cytoplasm of endothelial cells (adult and foetal brain) and in both the cytoplasm and nuclei of neurons (adult brain). The levels and subcellular distributions of these proteins in non-tumour tissues is reported Table 3. The levels of these four proteins were much higher in high-grade gliomas than in non-tumour tissues from the brain (Figure 1).

**Table 3:** Levels of EDN/RB, HJURP, p60/CAF-1 and PDLI4 proteins in adult and foetal brain, as determined by immunohistochemistry.

	**neuron**		**oligodendrocyte**		**astrocyte**		**endothelial cell**	
	Nuc.	Cyt.	Nuc.	Cyt.	Nuc.	Cyt.	Nuc.	Cyt.
**Adult brain (n=6**)								
p60/CAF-1	-	-	-	-	-	-	-	-
EDN/RB	-	+	-	-	-	-	-	+
HJURP	-	+	+	-	-	-	-	+
PDLI4	+	+	-	-	+	-	-	+
**Foetal brain (n=4**)								
p60/CAF-1	-	-	+	-	-	-	-	-
EDN/RB	-	+	+	-	-	-	-	+
HJURP	-	-	-	-	-	-	-	+
PDLI4	-	-	-	-	+	-	-	+

Nucleus: NucCytoplasm: Cyt

### EDN/RB, HJURP, p60/CAF-1 and PDLI4 levels distinguish anaplastic gliomas from glioblastomas

**Figure 2:**
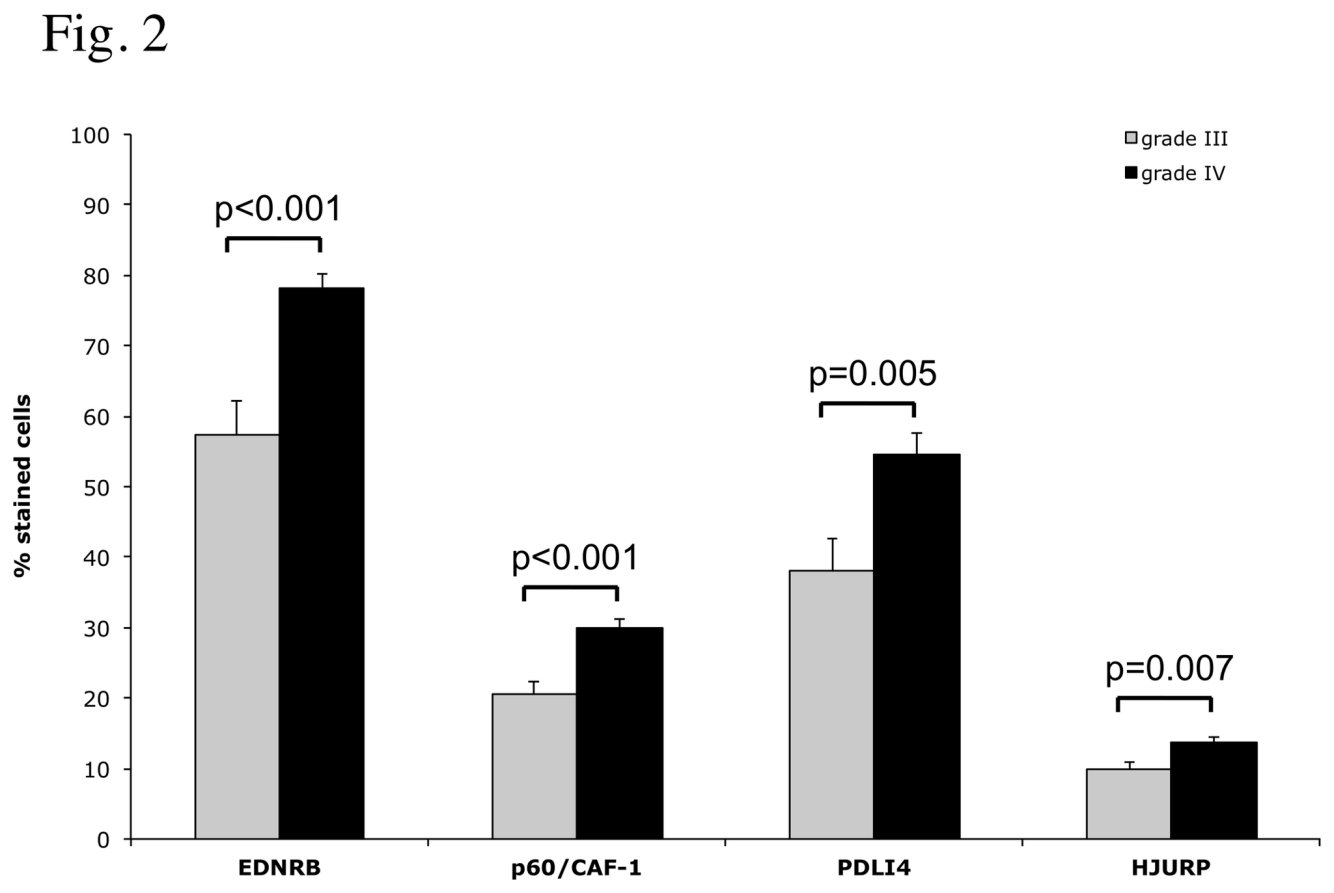
Immunohistochemical analyses of marker expression in grade III and grade IV gliomas. A statistical analysis of the difference in the percentage of positive cells for each marker between grade III (32 cases) and grade IV specimens (64 cases) is presented. P-values were obtained by applying a Wilcoxon rank sum test to each comparison.

In tumour samples, immunoreactivity was observed in the nucleus for p60/CAF-1 and HJURP, and in the cytoplasm for PDLI4 and EDN/RB. We looked for an association between the levels of each protein and clinical and pathological variables. No significant correlation was found between the level of expression of any of the proteins and age, sex or preoperative performance status. However, the percentage of displaying positive staining was significantly higher in glioblastomas than in grade III gliomas, for all the proteins tested (Figure 2, EDN/RB: p<0.001, p60/CAF-1: p<0.001, PDLI4: p=0.007 and HJURP: p<0.005). No differences were observed between AA and AO. These observations support the notion that the progression of high-grade gliomas is associated with increases in the levels of EDN/RB, HJURP, p60/CAF-1 and PDLI4.

### Correlation between protein abundances and mRNA levels

RT, Q-PCR gene expression data was available for 80 patients also included in our previous study^6^. These data were used to investigate the correlation between protein abundances and mRNA levels. There were no correlation (Pearson) between mRNA levels and corresponding IHC cell counts with the exception of the correlation between *CHAF1B* gene expression and p60/CAF-1 abundance (p-value = 0.02). For *EDNRB*, *HJURP* and *PDLIM4*, a higher expression of mRNA may suggest however a probable increased abundance of the corresponding protein. The comparison of protein abundances between high and low gene expressions (cut-off = 25^th^ percentile) show that *HJURP* and *PDLIM4* have similar expression patterns at both the mRNA and protein levels (p-value = 0.05 and p-value = 0.005, respectively). An opposite pattern was observed for *EDNRB* (p-value = 0.02). The expression of *EDNRB*, *HJURP* and *PDLIM4* genes were significantly different between glioblastomas and grade III gliomas (p<0.001, p=0.004 and p<0.001, respectively).

### EDN/RB, HJURP, p60/CAF-1 and PDLI4 levels are associated with patient prognosis

**Figure 3:**
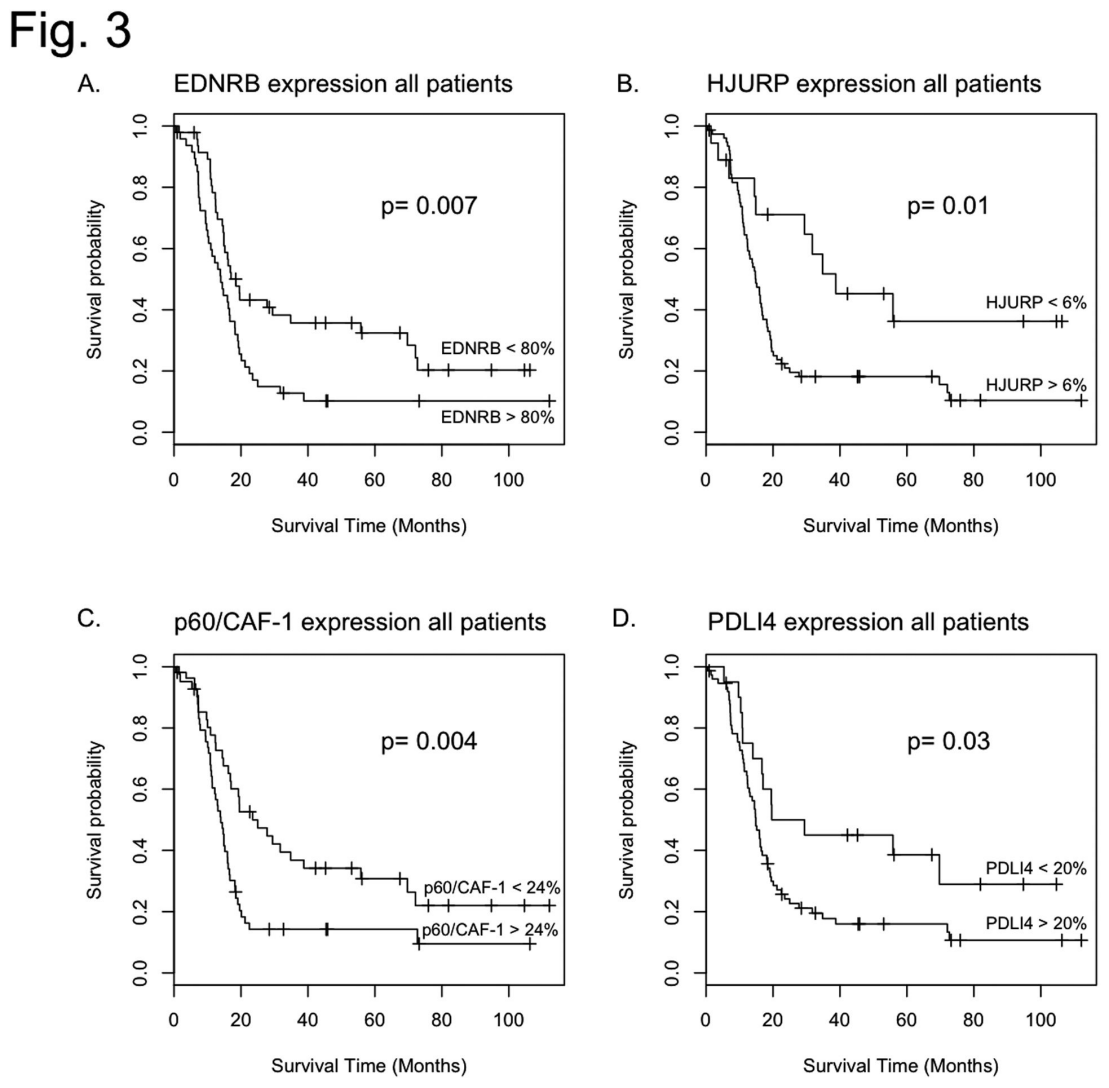
Overall survival analyses of molecular markers. Kaplan–Meier estimates of overall survival are presented for all markers (EDN/RB, HJURP, p60/CAF-1 and PDLI4), after subdivision of the cohort of patients into two groups (low and high risk of death) on the basis of the cut-off points defined by analyses of time-dependent ROC curves. Panel A. For the EDN/RB protein, median overall survival for low-risk patients was 18.5 months (95% CI, 14.9-69.7), whereas that for high-risk patients was 14 months (95% CI, 10.4-18.3) (P=0.007). Panel B. For the HJURP protein, the difference in overall survival between low-risk and high-risk patients is significant (P=0.01 with 38.8 months [95% CI, 29.4-12.5] versus 14.9 months [95% CI, 12.5 to 17], respectively). Panel C. For the p60/CAF-1 protein, the difference in overall survival between high-risk and low-risk patients was also significant (p=0.004, 14 months [95% CI, 11.4-16.2] versus 23.5 months [95% CI, 16.8-55.8], respectively). Panel D. For the PDLI4 protein, the difference is also significant (P=0.02, 14.9 months [95% CI, 13-18.2] versus 19.6 months [95% CI, 16.7-Inf]).

For each protein, we first analysed the percentage of immunoreactive cells before variable stratification. The results of the corresponding univariate survival analyses are presented in Table 1. These analyses revealed a strong association between overall survival and EDN/RB, p60/CAF-1 and HJURP levels (p<0.001, p<0.001 and p=0.002, respectively) but with PDLI4 levels (p=0.11). For each protein, we then assigned the patients to two groups (high expression levels and low expression levels) according to the cut-offs defined on the basis of the time-dependent ROC-curves. These cut-offs and associated performance values are summarised in Table 2. For each protein, log-rank tests and Kaplan-Meier analyses showed that the two groups of patients differed significantly in terms of overall survival (OS; see Figure 3 and Table 4): EDN/RB: p=0.007, p60/CAF-1: p=0.004, PDLI4: p=0.01 and HJURP: p=0.03. The median survival time for high levels of EDN/RB was 14 months (95% CI, 10.4-18.3), whereas that for low levels of EDN/RB was 18.5 months (95% CI, 14.9-69.7). The difference in OS between patients with high and low levels of the p60/CAF-1 protein was also significant (14 months [95% CI, 11.4-16.2] versus 23.5 months [95% CI, 16.8-55.8]). For the PDLI4 protein, median OS for patients with high levels of the protein was 14.9 months (95% CI, 13-18.2), whereas that for patients with low protein levels was 19.6 months (95% CI, 16.7-Inf), this difference being significant. Stratification on the basis of HJURP protein levels identified a group of long-term survivors (38.8 months [95% CI, 29.4-12.5]). Multivariate survival analyses including age and treatment indicated that the levels of the four proteins were prognostic factors independent of age and treatment (Table 5). We also carried out multivariate analyses including WHO grade. In these analyses, the levels of none of the four proteins were significant, confirming that their prognostic values depend on malignancy grade.

**Table 4:** Immunohistochemical results for EDNRB, HJURP, p60/CAF-1 and PDLI4, and univariate survival analysis.

Marker		No. Patients	Age--no.		Stage		Survival -- months		Univariate
			≤50 years	>50 years	IV	III	Median	95% CI	analysis
EDNRB									0.007
	<80%	48	16	32	24	24	18.5	14.9-69.7	
	>80%	48	12	36	40	8	14	10.4-18.3	
p60/CAF-1									0.004
	<24%	41	17	24	18	23	23.5	16.8-55.8	
	>24%	55	11	44	46	9	14	11.4-16.2	
PDLI4									0.03
	<20%	21	5	16	8	13	19.6	16.7-Inf	
	>20%	75	23	52	56	19	14.9	13-18.2	
HJURP									0.01
	<6%	19	9	10	8	11	38.8	29.4-12.5	
	>6%	77	19	58	56	21	14.9	12.5-17	

**Table 5:** Multivariate analysis of EDNRB, HJURP, p60/CAF-1 and PDLI4.

	P Value of covariate in cox models		
	Marker	Age	Treatment
EDNRB	0.007	-	-
EDNRB+Age	0.009	0.04	-
EDNRB+Treatment	0.01	-	0.05
EDNRB+Age+Treatment	0.01	0.09	0.1
HJURP	0.01	-	-
HJURP+Age	0.01	0.04	-
HJURP+Treatment	0.01	-	0.04
HJURP+Age+Treatment	0.01	0.1	0.1
p60/CAF-1	0.004	-	-
p60/CAF-1+Age	0.02	0.14	-
p60/CAF-1+Treatment	0.003	-	0.06
p60/CAF-1+Age+Treatment	0.01	0.3	0.09
PDLI4	0.03	-	-
PDLI4+Age	0.03	0.03	
PDLI4+Treatment	0.02	-	0.04
PDLI4+Age+Treatment	0.03	0.09	0.09

### High predictive power of the combined study of EDN/RB, p60/CAF-1 and HJURP levels

Based on these results, we consider EDN/RB, p60/CAF-1 and HJURP as the most relevant markers for predicting HGG prognosis. The prognostic value of the risk factor defined on the abundances of these proteins was evaluated. The resulting stratification gave 62 patients in the high-risk group (10 grade III and 52 grade IV) and 33 patients in the low-risk group (22 grade III and 11 grade IV). These risk groups differed significantly in terms of overall survival (p<0.001), with median survival times of 14 months (95% CI, 11.4-16.2) for the high-risk group and 34.8 months (95% CI, 19.5-Inf) for the low-risk group. After adjustment for age and treatment, multivariate analysis confirmed that this criterion was an independent negative prognostic marker (hazard ratio = 2.22; 95% CI, 1.25 to 3.97, p=0.007). By comparing two multivariate models, both including age, treatment, and WHO grade, one with and one without the risk criterion, we showed that this criterion was additional to known clinical factors (p=0.04). *IDH1* mutational status and its association with outcome were assessed in 75 of our patients (10 tumours with mutations vs 65 non-mutated tumours, p<0.001). The association of *MGMT* methylation status with overall survival was also observed for the 76 patients with available measurements (hazard ratio = 0.98; 95% CI, 0.97 to 0.99, p=0.006 adjusted for WHO grade). In the subset of patients with both *MGMT* and *IDH1* statuses available (n= 72), we evaluated a multivariate model including age, treatment, *MGMT* methylation status, *IDH1* mutational status, and the protein risk criterion. This analysis showed that *IDH1* mutational status and the risk criterion were independent prognostic factors in this model (p = 0.02 and p = 0.02, respectively). Comparison between the 4-gene mRNA signature and the protein risk criterion was performed in a multivariate analysis. Both signatures were associated with overall survival: 4-gene signature (hazard ratio = 2.90; 95% CI, 1.56 to 5.39, p=0.0008) and protein signature (hazard ratio = 1.90; 95% CI, 1.06 to 3.41, p=0.03).

## Discussion

In this study, we analysed the levels of the proteins encoded by four genes that we previously defined as a prognostic risk panel in a meta-analysis of microarray data^6^. Mean levels of the EDN/RB, HJURP, p60/CAF-1 and PDLI4 proteins were found to be significantly higher in grade IV gliomas than in grade III gliomas. The upregulation of these proteins was significantly associated with a pour outcome of HGGs. Our results also suggest that the levels of these proteins are linked to patient outcome. We also demonstrated that a combination of the immunohistochemical results for EDN/RB, p60/CAF-1 and HJURP constituted an important and independent source of prognostic information for patients with HGGs.

This work and our previous results show a similar pattern for *CHAF1B*, *PDLIM4* and *HJURP* expression, at both the mRNA and protein levels, but with opposite patterns for EDN/RB. We show here that high levels of EDN/RB protein are correlated with a poor prognosis, whereas we previously showed that high levels of mRNA were protective. Mismatches between protein and mRNA levels have been studied in several biological processes and, in most cases, only a weak correlation has been found between protein and mRNA abundances. Many studies have suggested that external factors and regulatory mechanisms may affect the relationships between mRNAs and proteins. Several biological factors influencing this correlation have been identified, including post-transcriptional modifications, but methodological constraints may also affect the relationship between mRNA and protein levels [8].

Very few studies of *EDN/RB* overexpression in gliomas have been published. Naidoo and coworkers were the first to describe endothelin B receptor overexpression in low-grade astrocytomas [9]. Anguelnova et al. highlighted the occurrence of EDN/RB overexpression in a series of both low- and high-grade gliomas and also reported the presence of this receptor in the endothelial cells of capillaries in normal brain parenchyma. However, the distribution of positive cells and the intensity of immunostaining were highly variable, in both infiltrated and solid tumour tissue. This previous study also showed that tumour cells displayed variable labelling of the nucleus and or cytoplasm [10]. EDN/RB expression has also been reported in other malignant cancers, such as malignant melanomas [11], and carcinomas of the bladder [12], ovary [13], breast [14] and lung [15]. In malignant melanomas, EDN/RB expression increases with the degree of invasion. Immunohistochemical staining showed that EDN/RB immunoreactivity was stronger in primary malignant melanomas than in dysplastic naevi, whereas metastatic melanomas were more strongly stained than primary malignant melanomas. These data suggest that EDN/RB is involved in tumour progression in malignant melanomas [11].

This work highlighted the tight control of *p60/CAF-1* expression in HGGs. The protein was not present in normal mature cerebral parenchyma and had a tumour expression profile similar to that of Ki67/MIB1. Both these proteins reflect the proliferation activity of the tissue sample, demonstrating the value of studying p60/CAF-1 levels for neuropathological diagnosis. The detection of p60/CAF-1, even at low levels, implies that a proliferation process is occurring. Indeed, p60/CAF-1 was recently proposed as a new marker of proliferation and prognosis. This protein was found to be overproduced in a series of human cancers, and its levels were closely associated with the biological aggressiveness of the tumour. Mascalo et al. showed that there was a gradient of p60/CAF-1 overproduction between benign naevi and malignant melanomas and that the levels of this protein increased significantly between radial (intraepithelial) growth and vertical (invasive) growth in malignant melanomas. These results suggest that p60/CAF-1 levels are likely to give accurate predictions of prognosis in neoplastic processes [16]. CAF-1/p60 expression levels have also been proposed as a new tool for defining the behaviour of carcinomas of the tongue [17], prostate [18] or breast [19].

PDLI4 protein levels have not been studied in previous investigations. The PDZ and LIM domain protein (PDLI4) regulates actin stress fibre turnover. It is thought to act as a tumour suppressor in myeloid diseases [20] and prostate cancer [21] and to have oncogenic potential in breast carcinoma [22], but its role remains unclear.

The Holliday Junction Recognition Protein (HJURP) is a centromeric protein that was recently shown to be essential for regulating centromeric chromatin assembly during the cell cycle [23,24] and for chromosomal stability in immortalised cancer cells [25]. HJURP mRNA levels were identified as a predictive marker of prognosis and of sensitivity to radiotherapy in breast cancer [26]. Recently, Valente et al. confirmed that modulation of HJURP levels is correlated with glioblastoma cells survival [27]. The concordance between HJURP mRNA and protein levels and their correlation with patient outcome suggest that HJURP is a potentially useful marker for diagnosis in neuropathology.

We also evaluated the relevance of multiple protein scoring. We showed that a protein scoring system based on HJURP, p60/CAF-1 and EDNR/B levels was an important source of prognostic information for patients with HGGs. This risk criterion has the advantage of being feasible to calculate for tumour samples embedded in paraffin and not requiring the use of frozen tissue — one of the major limitations of studies of these tumours in clinical practice. Further multicentre evaluation of these new biomarkers is required, but our results suggest that they may consistently increase the precision of HGG diagnosis.

Further studies are required to determine the value of these findings for clinical and therapeutic management. However, these new biomarkers, specifically associated with tumour grade (III vs IV) and correlated with patient outcome may be considered promising.

## Supporting Information

Figure S1 Time-dependent AUC plots and ROC curves used for optimal cut-off selection.
(TIFF)
